# Supplementary material for: Matere Bonds vs. Multivalent Halogen and Chalcogen Bonds: Three Case Studies
Source: Molecules. 2022 Oct 5;27(19):6597. doi: 10.3390/molecules27196597 (PMC9571139; doi:10.3390/molecules27196597)
Supplement: Supplementary file 1 [file molecules-27-06597-s001.zip › molecules-1949651-supplementary.pdf]

Supplementary Material for manuscript

Matere bonds *vs* multivalent halogen and chalcogen bonds: three case studies

Rosa M. Gomila[1], and Antonio Frontera[1,*]

[1] Universitat de les Illes Balears, Crta de Valldemossa km 7.5, 0722 Palma de Mallorca (Baleares), SPAIN
* Correspondence: toni.frontera@uib.es

Cartesian Coordinates

HETRUT (opposite to O-Re)

Si	-2.1816788	-0.6697500	4.9737811
O	-2.1237179	-0.5789841	3.2832367
Re	-0.7969309	-0.2625058	2.0580288
O	-0.1895637	1.3016652	2.3062257
O	-1.4361758	-0.4077736	0.4925105
O	0.4571911	-1.3825852	2.2967184
C	-3.4773506	-1.9003583	5.4108024
C	-2.4588284	1.0521060	5.5805631
O	-0.7101180	-1.2225968	5.4031177
Si	0.8563118	-1.1161104	5.8392020
O	0.8178652	-1.1155942	7.5344852
Re	-0.3557648	-0.5290886	8.8135440
O	-1.8932153	-1.1953535	8.5285484
O	-0.4786230	1.1618714	8.7274639
O	0.2016180	-0.9912620	10.3454962
C	1.7702151	-2.6250400	5.3191075
C	1.5903736	0.4994511	5.3310763
Si	2.1816788	0.6697500	-4.9737811
O	2.1237179	0.5789841	-3.2832367
Re	0.7969309	0.2625058	-2.0580288
O	0.1895637	-1.3016652	-2.3062257
O	1.4361758	0.4077736	-0.4925105
O	-0.4571911	1.3825852	-2.2967184
C	3.4773506	1.9003583	-5.4108024

C	2.4588284	-1.0521060	-5.5805631
O	0.7101180	1.2225968	-5.4031177
Si	-0.8563118	1.1161104	-5.8392020
O	-0.8178652	1.1155942	-7.5344852
Re	0.3557648	0.5290886	-8.8135440
O	1.8932153	1.1953535	-8.5285484
O	0.4786230	-1.1618714	-8.7274639
O	-0.2016180	0.9912620	-10.3454962
C	-1.7702151	2.6250400	-5.3191075
C	-1.5903736	-0.4994511	-5.3310763
H	-2.8013424	2.6050957	-5.6781795
H	-1.2848531	3.5228338	-5.7076039
H	-1.7813452	2.6801612	-4.2276674
H	-2.6189269	-0.5855709	-5.6892338
H	-1.5998963	-0.6028536	-4.2432531
H	-1.0178439	-1.3331217	-5.7463177
H	3.5374482	1.9884118	-6.4985368
H	3.2331001	2.8808926	-4.9968153
H	4.4548545	1.5961614	-5.0305531
H	1.7065756	-1.7330608	-5.1735909
H	3.4428020	-1.4134447	-5.2726091
H	2.4069627	-1.0990019	-6.6712571
H	-3.4428020	1.4134447	5.2726091
H	-4.4548545	-1.5961614	5.0305531
H	-1.7065756	1.7330608	5.1735909
H	-2.4069627	1.0990019	6.6712571
H	-3.5374482	-1.9884118	6.4985368
H	1.0178439	1.3331217	5.7463177
H	-3.2331001	-2.8808926	4.9968153
H	1.5998963	0.6028536	4.2432531
H	2.6189269	0.5855709	5.6892338
H	1.7813452	-2.6801612	4.2276674
H	1.2848531	-3.5228338	5.7076039
H	2.8013424	-2.6050957	5.6781795

HETRUT (opposite to O=Re)

Si	-1.5453810	0.4494899	-5.0353027
O	-0.7027779	0.0473368	-6.4431362
Re	0.5594043	-1.1278998	-7.0613779
O	2.1015409	-0.4445026	-6.8818885
O	0.2701915	-1.4585086	-8.6984000
O	0.4915496	-2.5677923	-6.1623284
C	-3.3368873	0.4413106	-5.4555979
C	-0.8631420	2.0720719	-4.4680197
O	-1.2046702	-0.7527525	-3.9878982
Si	-0.2220825	-1.3313249	-2.8205347
O	-0.5512661	-0.3208927	-1.4832718
Re	-1.9533209	0.6259438	-0.7870471
O	-3.0865690	0.9299381	-2.0132600
O	-1.3976985	2.1049236	-0.1605039
O	-2.7043600	-0.2581825	0.4494228
C	-0.7407856	-3.0362591	-2.3680033
C	1.5607398	-1.1075830	-3.2233638
Si	1.5453810	-0.4494899	5.0353027
O	0.7027779	-0.0473368	6.4431362
Re	-0.5594043	1.1278998	7.0613779
O	-2.1015409	0.4445026	6.8818885
O	-0.2701915	1.4585086	8.6984000
O	-0.4915496	2.5677923	6.1623284
C	3.3368873	-0.4413106	5.4555979
C	0.8631420	-2.0720719	4.4680197
O	1.2046702	0.7527525	3.9878982
Si	0.2220825	1.3313249	2.8205347
O	0.5512661	0.3208927	1.4832718
Re	1.9533209	-0.6259438	0.7870471
O	3.0865690	-0.9299381	2.0132600
O	1.3976985	-2.1049236	0.1605039
O	2.7043600	0.2581825	-0.4494228
C	0.7407856	3.0362591	2.3680033
C	-1.5607398	1.1075830	3.2233638

H	-3.5623223	1.1849265	-6.2231947
H	-3.6401401	-0.5403650	-5.8257540
H	-3.9235181	0.6720966	-4.5633126
H	-0.9318999	2.8155890	-5.2655868
H	0.1876873	1.9826181	-4.1823772
H	-1.4263001	2.4480582	-3.6106246
H	1.8036730	-0.0689637	-3.4563875
H	1.8229192	-1.7289862	-4.0829131
H	-1.8036147	-3.0677716	-2.1171959
H	-0.5680571	-3.7084717	-3.2122930
H	-0.1655354	-3.3918944	-1.5110753
H	2.1834274	-1.4149226	-2.3817370
H	0.5680571	3.7084717	3.2122930
H	0.1655354	3.3918944	1.5110753
H	1.8036147	3.0677716	2.1171959
H	3.9235181	-0.6720966	4.5633126
H	3.5623223	-1.1849265	6.2231947
H	3.6401401	0.5403650	5.8257540
H	0.9318999	-2.8155890	5.2655868
H	1.4263001	-2.4480582	3.6106246
H	-0.1876873	-1.9826181	4.1823772
H	-1.8229192	1.7289862	4.0829131
H	-2.1834274	1.4149226	2.3817370
H	-1.8036730	0.0689637	3.4563875

WABPAR

Se	0.9740838	-1.4940238	-0.2571223
O	2.3791644	-2.5390417	-1.5606468
C	3.7905664	-0.7850592	-2.4301394
H	3.5264550	-1.0480729	-3.4558182
H	4.8115905	-0.3969920	-2.4212695
H	3.1164779	-0.0008461	-2.0881615
C	3.7189696	-1.9933281	-1.5350520
H	4.3879084	-2.7844585	-1.8841355
H	3.9717035	-1.7550310	-0.4987400

C	2.8250946	-4.5153924	-0.1385357
H	3.8860045	-4.2989997	-0.0000753
H	2.7006169	-5.6004711	-0.1323828
H	2.2736529	-4.1010848	0.7060000
C	2.3014118	-3.9765043	-1.4467045
H	2.8511548	-4.3769811	-2.3039780
H	1.2442057	-4.2041257	-1.5731474
O	-0.1415846	-2.6216803	-0.3592691
O	0.8665281	-0.1956274	-1.1801702
O	1.9388209	-1.4547290	1.0078332
Se	-0.9732869	1.4947031	0.2586375
O	-2.3793659	2.5391616	1.5611213
C	-3.7904511	0.7846033	2.4295863
H	-3.5267144	1.0476151	3.4553703
H	-4.8112950	0.3960781	2.4204611
H	-3.1159756	0.0007720	2.0875918
C	-3.7190305	1.9931293	1.5348567
H	-4.3880574	2.7840532	1.8841881
H	-3.9715408	1.7552765	0.4983853
C	-2.8253606	4.5149755	0.1378405
H	-3.8861804	4.2984765	-0.0010988
H	-2.7010732	5.6000739	0.1319341
H	-2.2735734	4.1009206	-0.7065653
C	-2.3021339	3.9765769	1.4464445
H	-2.8525867	4.3771791	2.3031684
H	-1.2450534	4.2046060	1.5732776
O	0.1419114	2.6229053	0.3605586
O	-0.8649924	0.1965827	1.1819800
O	-1.9380650	1.4547605	-1.0062231

KUCRAD

Cl	1.8956611	0.1025675	-0.9299427
O	1.4895959	1.2464611	-1.6350026
O	1.5256301	0.0900337	0.4330344
O	1.6413172	-1.1202813	-1.5889758

O	3.4968815	0.2833111	-0.9424671
C	5.6514947	-0.3518655	-0.1176482
C	5.6977574	0.8052668	0.8866267
H	5.1260940	1.6536022	0.4988779
H	5.2181498	0.4957967	1.8229408
C	7.1459957	1.2152951	1.1464182
H	7.1627600	2.0437421	1.8617517
C	7.7910607	1.6565751	-0.1674994
H	7.2555546	2.5198198	-0.5772258
H	8.8245862	1.9727488	0.0103196
C	7.7634157	0.4996903	-1.1667895
H	8.2223520	0.8161214	-2.1087948
C	6.3149480	0.0891016	-1.4276049
H	6.2790774	-0.7339678	-2.1519612
H	5.7574452	0.9240104	-1.8620552
C	6.4356555	-1.5389741	0.4581862
H	5.9671959	-1.8770267	1.3900820
H	6.4033426	-2.3805885	-0.2439602
C	7.8853667	-1.1313871	0.7197709
H	8.4289828	-1.9883064	1.1300386
C	7.9156228	0.0251233	1.7189001
H	8.9519168	0.3118336	1.9277467
H	7.4709756	-0.2878871	2.6700568
C	8.5340477	-0.6887791	-0.5919537
H	8.5379163	-1.5178523	-1.3087120
H	9.5788919	-0.4101558	-0.4172636
C	4.2274704	-0.8131043	-0.3469786
H	4.1849571	-1.6631821	-1.0330867
H	3.7539816	-1.0795693	0.6026836
Cl	-1.8956611	-0.1025675	0.9299427
O	-1.4895959	-1.2464611	1.6350026
O	-1.5256301	-0.0900337	-0.4330344
O	-1.6413172	1.1202813	1.5889758
O	-3.4968815	-0.2833111	0.9424671
C	-5.6514947	0.3518655	0.1176482

C	-5.6977574	-0.8052668	-0.8866267
H	-5.1260940	-1.6536022	-0.4988779
H	-5.2181498	-0.4957967	-1.8229408
C	-7.1459957	-1.2152951	-1.1464182
H	-7.1627600	-2.0437421	-1.8617517
C	-7.7910607	-1.6565751	0.1674994
H	-7.2555546	-2.5198198	0.5772258
H	-8.8245862	-1.9727488	-0.0103196
C	-7.7634157	-0.4996903	1.1667895
H	-8.2223520	-0.8161214	2.1087948
C	-6.3149480	-0.0891016	1.4276049
H	-6.2790774	0.7339678	2.1519612
H	-5.7574452	-0.9240104	1.8620552
C	-6.4356555	1.5389741	-0.4581862
H	-5.9671959	1.8770267	-1.3900820
H	-6.4033426	2.3805885	0.2439602
C	-7.8853667	1.1313871	-0.7197709
H	-8.4289828	1.9883064	-1.1300386
C	-7.9156228	-0.0251233	-1.7189001
H	-8.9519168	-0.3118336	-1.9277467
H	-7.4709756	0.2878871	-2.6700568
C	-8.5340477	0.6887791	0.5919537
H	-8.5379163	1.5178523	1.3087120
H	-9.5788919	0.4101558	0.4172636
C	-4.2274704	0.8131043	0.3469786
H	-4.1849571	1.6631821	1.0330867
H	-3.7539816	1.0795693	-0.6026836
